# A score based likelihood ratio framework for deepfake image identification in forensic science

**DOI:** 10.1038/s41598-026-42176-w

**Published:** 2026-03-05

**Authors:** Tianli Guo, Jisong Li, Yunqi Tang

**Affiliations:** School of Criminal Investigation, People’s Public Security University of China, Beijing, 100038 China

**Keywords:** Deepfake images, Detection, Score, LR, Identification, Engineering, Mathematics and computing

## Abstract

This paper proposes a score-based likelihood ratio system for forensic identification of deepfake images, addressing challenges in digital media identification due to rapid deepfake development. Built on the FaceForensics + + dataset, the system prevents data leakage via video-level splits (training, validation, selection, calibration, and test sets). Among six candidate models, the Capsule detector demonstrates the most robust performance (AUC = 0.983). Score distributions of real and fake samples are modeled using kernel density estimation, with optimal bandwidths selected through a two-stage search (real: 0.004, fake: 0.003). Extreme LR values are bounded using the empirical lower and upper bounds method (− 2.3634 to 1.9933), and PAV calibration is applied to optimize the calibration performance of the LR system. On the FF + + test set, the system exhibits favorable performancewith forensic practice expectations: low misleading evidence rates (RMEP = 0.069, RMED = 0.092), good error control (EER = 0.0804), and reduced decision loss after calibration (the cost log-likelihood ratio from 0.2899 to 0.1625). Generalization tests on five unseen datasets (Celeb-DF-v1/v2, DFDCP_methodA/B, UADFV) yield AUCs between 0.621 and 0.783—highest on UADFV (0.783), stable on DFDCP, weaker on Celeb-DF. The results show that at the moment, the technique shows potential for forensic-oriented deepfake identification, but requires further validation across diverse real-world scenarios before practical forensic application.

## Introduction

Deepfake images have undergone rapid development, driven by advances in deep learning technologies such as GANs and autoencoders. Applications including FaceSwap, FaceReenactment, Zao, and FaceApp enable sophisticated manipulations such as facial replacement and expression alteration. As deepfake technology continues to evolve, the resulting deepfake images and videos have become increasingly realistic^1^, posing significant challenges for deepfake detection via visual inspection alone.

These capabilities raise significant concerns, particularly when used to generate non-consensual explicit content involving public figures or to fabricate statements by political leaders—threats that undermine information security and challenge judicial integrity. In light of growing doubts about the reality of digital media, forensic experts are increasingly called upon to perform verification and analysis^2^. This gives rise to a critical forensic task: examining suspect images to determine whether they are real or deepfake. Such determinations not only establish the veracity of the visual content but also serve as crucial evidentiary support in criminal investigations, courtroom proceedings, and legal defenses^3^.

**Fig. 1:**
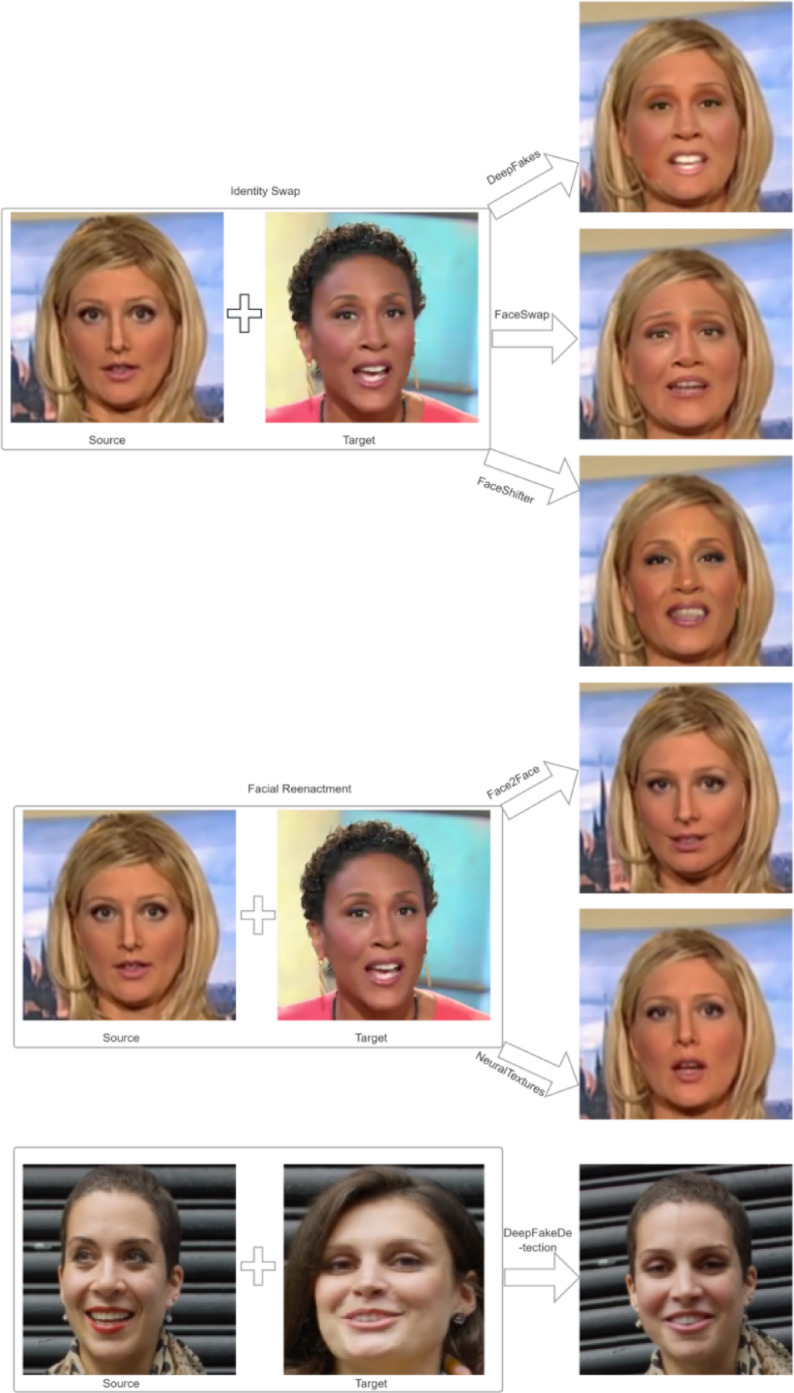
Real and deepfake images.

This study used the FaceForensics++ (FF++) dataset due to its academic authority and widespread recognition in deepfake research. The dataset covers major real-world deepfake techniques, enabling comprehensive analysis of diverse manipulation scenarios. It provides high-quality images and detailed annotations, ensuring reliable and reproducible results. FF + + includes multiple generation methods and multi-resolution samples, supporting the development of a generalizable forensic framework. Its structure allows systematic evaluation across different deepfake types, facilitating model optimization and practical application^4^. Figure 1 presents representative examples of images from the FF + + dataset.

**Fig. 2:**
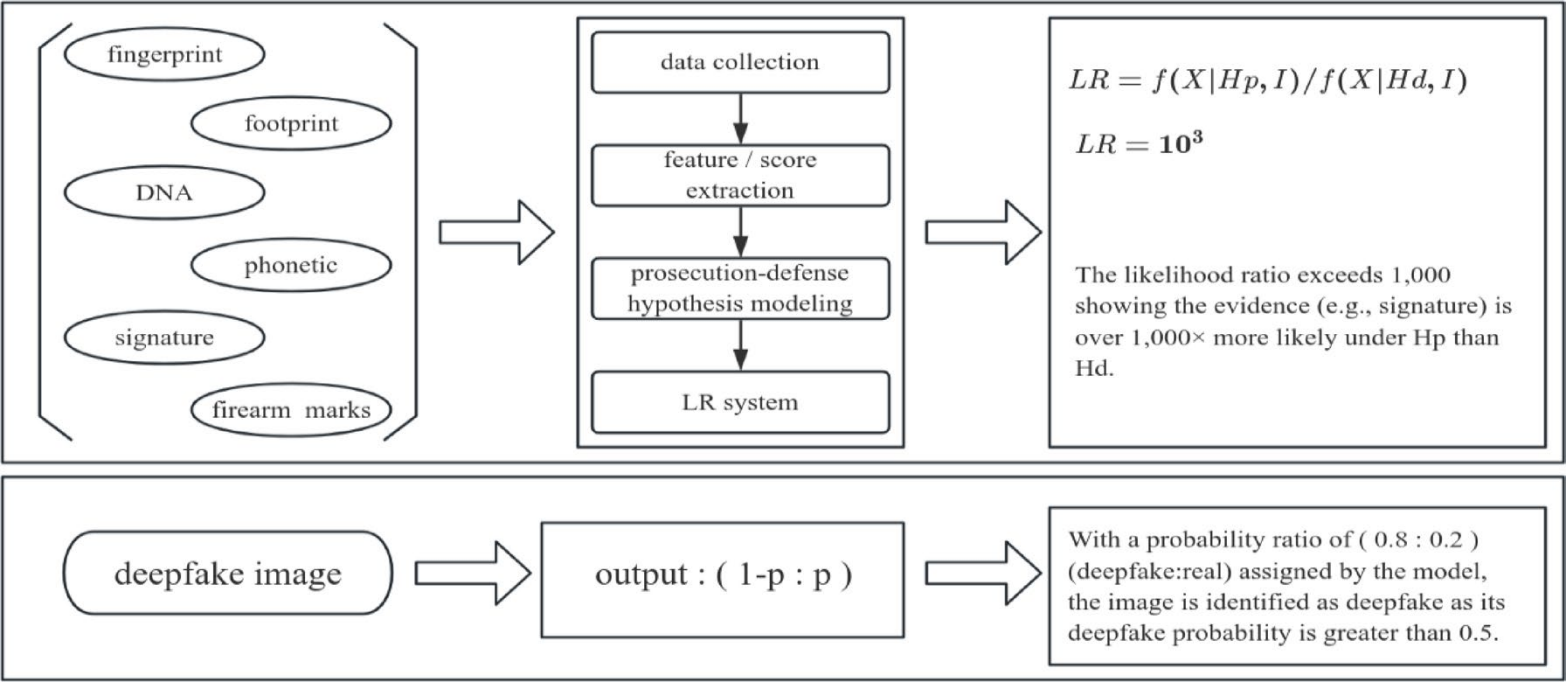
Deepfake detector outputs vs. forensic evidence practice expectations: current gaps.

Deepfake images are increasingly used in criminal activities—for example, fraudsters employ face-swapping to create deceptive videos for telecom scams^5^. In such cases, courts must not only determine whether video evidence is forged but also assess its credibility quantitatively. However, current deepfake detection methods have not yet fully addressed the practical needs and reliability expectations for applications in forensic context^6–13^. Most existing deepfake detectors produce only binary classifications—labeling images as “real” or “fake”—and critically lack quantitative measures of evidential strength, such as likelihood ratio (LR), which are essential for assessing evidence reliability in forensic contexts. Their uncalibrated confidence scores cannot serve as reliable probabilities or support valid comparisons between competing hypotheses. As shown in Fig. 2, forensic evidence (e.g., signature) outputs a LR to quantify evidential strength (e.g., LR = 10³ measures support for hypotheses)^14^. In contrast, current deepfake detectors only give binary “real/fake” classifications (e.g., 0.8:0.2 ratio) without quantifying evidence strength. While they answer “Is it fake?“, they fail to address the forensic question: “How strong is the evidence?“—this highlights a critical gap between existing deepfake detection tools and the practical expectations for evidential assessment in forensic contexts.

To bridge this gap, we convert deepfake detection outputs into continuous scores. By comparing a test image’s score against real and fake score distributions, we compute a LR that evaluates the two key judicial hypotheses: the prosecution’ s (Hp: forged) and the defense’ s (Hd: authentic). To improve upon existing LR methods and align with forensic practice expectations, we propose three innovations. First, we introduce a model selection criterion tailored to forensic scenarios, prioritizing both overall accuracy and balanced performance across deepfake subtypes, reducing blind spots from overreliance on single metrics. Second, we construct a LR model of deepfake images using detector scores to comprehensively characterize their features. Third, we apply empirical lower and upper bounds (ELUB) adjustment to a deepfake LR framework, limiting risks from tail-data extrapolation. Together, these advances convert model outputs into relatively interpretable and scientifically informed indicators of evidential support, aligning automated detection with courtroom practice.

The paper is structured as follows. Section 2 reviews state-of-the-art deepfake techniques, detection methods, and LR applications in facial verification. Section 3 details the proposed framework, including dataset partitioning, experimental setup, and implementation of the three innovations. Section 4 presents results and discussion of the LR system. Section 5 concludes with summary remarks.

## Related work

### Deepfake techniques

Facial swapping, facial reenactment, facial editing, and facial generation are among the most widely studied deepfake techniques. These methods manipulate facial images or videos using diverse algorithms and processing pipelines, posing significant risks in areas such as misinformation dissemination and identity fraud. Facial swapping involves replacing the source face in one image or video with a target face from another, enabling the target individual to appear as if they are present in the source context. This technique can achieve a high degree of visual realism by accurately preserving facial geometry, proportions, texture, and skin tone^15^. Facial reenactment aims to transfer the dynamic expressions of a person in a source video onto the face of a target individual in a different video, while preserving the latter’s unique facial identity. This process can transform a neutral or solemn expression into a smile, surprise, or sorrow, thereby creating highly convincing deepfake performances^16^. Facial editing focuses on localized modifications of facial features, such as altering mouth shape and size or adjusting eye expressions, to modify specific regions of the face. These changes may range from subtle enhancements—such as increasing eye brightness or expressiveness—to more pronounced alterations, like exaggerating lip size^17^. In contrast, facial generation synthesizes entirely novel facial images or videos. The resulting faces do not correspond to any real individual but are algorithmically generated, often featuring customizable attributes such as hairstyle, facial structure, skin color, and stylistic elements^18^.

### Deepfake detection

Existing deepfake detection methods target specific artifacts of different generation techniques. A multi-level wavelet transform and vision transformer approach detects frequency anomalies, shows potential for detecting high-fidelity deepfakes from Diffusion models or advanced GANs that have subtle texture flaws invisible in the spatial domain^19^. An attention-based data augmentation method improves robustness, making it suitable for low-quality face-swapped deepfakes with inconsistent facial details^20^. A mutual attention Transformer identifies abnormal visual patterns caused by facial expression manipulation (e.g., Face2Face)^21^. SCNet (denoising) and UTMCR (dehazing) enhance image quality, aiding detection in noisy or hazy conditions common in low-cost forgeries^22,23^. FFFN improves temporal consistency analysis to detect motion incoherences in real-time face swap or lip-sync videos^24^. FSAD-Net uses spatial attention for dehazing, helping identify deepfakes with warped facial structures^25^.

Deepfake detectors fall into three main categories aligned with distinct artifact types. First, CNN-based binary classifiers use end-to-end learning to distinguish real from fake images, especially effective for early-stage deepfakes with obvious visual differences, without needing prior knowledge of specific forgery patterns^26–31^. Second, spatial-domain detectors analyze geometric misalignments (e.g., mismatched eye spacing), structural inconsistencies, and texture discontinuities—hallmarks of face-swapping methods like DeepFake and FaceSwap—directly exposing their core spatial flaws^32–36^. Third, frequency-domain detectors apply Fourier or wavelet transforms to uncover unnatural periodic signals or compression artifacts in high-fidelity deepfakes (e.g., from Diffusion models, StyleGAN3), which appear realistic spatially but show abnormal frequency patterns, thus overcoming the limits of spatial methods^37–42^.

### Score-based LR

The score-based LR has been widely applied in the field of facial image verification^43^. Paulo Max Gil Inunencio Reis et al. proposed a facial similarity scoring method using deep convolutional neural networks. The method computes similarity scores but classifies videos as real or fake by comparing them to a fixed threshold, staying within a binary framework. It does not enable quantitative assessment of evidential strength and remains limited to qualitative “real/fake” decisions^44^. Andrea Macanulla Rodriguez et al. introduced a calibration framework grounded in the score-based LR, computing calibrated LRs to evaluate the reliability of an automated facial comparison system^45^. Jin et al. investigated the relation of AI-generated images using the score-based LR method, further validating its utility in distinguishing deepfakes from real facial content^46^.

## Materials and methods

### Materials

#### Datasets

The FF + + dataset includes four main deepfake methods—Deepfakes, Face2Face, FaceSwap, and Neural Textures—with 1,000 videos each. The FaceShifter method, which uses HEAR-Net to better handle occluded facial regions, was later added to generate additional deepfakes for the same 1,000 real videos^47^. The dataset also incorporates Google and Jigsaw’s Deep Fake Detection Dataset, adding over 3,000 deepfake videos featuring 28 actors in diverse scenarios^48^.

**Table 1:** Dataset division.

Dataset	Training		Validation		Select		Calibration		Test	
	Vid	Frm	Vid	Frm	Vid	Frm	Vid	Frm	Vid	Frm
real	499	15,958	99	3166	99	3167	199	6367	103	3291
Deepfakes	499	15,867	99	3165	99	3164	199	6361	103	3286
Face2Face	499	15,956	99	3168	99	3168	199	6364	103	3293
FaceSwap	500	15,973	100	3196	100	3196	200	6389	100	3200
NeuralTextures	499	15,944	99	3164	99	3166	199	6365	103	3292
FaceShifter	499	15,948	99	3168	99	3166	199	6362	103	3280
DeepFakeDetection	1533	46,107	306	9204	306	9053	613	18,262	308	9174

The FF + + dataset is partitioned at the video level into five non-overlapping subsets—training, validation, selection, calibration, and test sets—in a 5:1:1:2:1 ratio. The training set trains the model; the validation set monitors performance for hyperparameter tuning; the selection set selects the optimal detector for score collection; the calibration set fits scores and constructs the LR system; and the test set provides an independent evaluation of final performance. This video-level partitioning strategy effectively prevents data leakage and reduces overfitting by eliminating frame-level dependencies associated with individual videos, thereby ensuring reliable and unbiased model evaluation. During preprocessing, a fixed number of frames are extracted from each video. Facial regions are aligned and cropped using affine transformations based on facial landmarks^49^, producing standardized face images and converting the dataset from video to image format. Table 1 shows the division results of the dataset in the study, where “Vid” represents “videos” and “Frm” represents “frames”.

#### Experimental configuration

The experiments were conducted on the Ubuntu 18.04 operating system, equipped with an NVIDIA GeForce RTX 3090 graphics processing unit (GPU). The experimental environment utilized Python version 3.7.12 and PyTorch version 1.13.1 + cu117.

For data preprocessing, frames were extracted from videos for training, validation, and testing purposes. All images were resized to a uniform resolution of 256 × 256 pixels. Image pixel values were normalized using a mean of [0.5, 0.5, 0.5] and a standard deviation of [0.5, 0.5, 0.5] across the three color channels. To ensure label consistency across datasets, a unified label mapping scheme was applied: deepfake samples were assigned a label of 1, while real samples were labeled as 0. Data loading was performed using eight parallel worker processes to enhance efficiency. The batch size was set to 100 for all phases.

The optimization strategy employed the Adam optimizer with an initial learning rate of 8 × 10^− 5^, β_1_ = 0.9, β_2_ = 0.999, and ε = 1 × 10^− 8^. Weight decay regularization (λ = 0.0005) was applied to mitigate overfitting. The Capsule_loss function was used as the objective function, and model performance was evaluated using the area under the ROC curve (AUC). Training was conducted for a total of 10 epochs. The model checkpoint was saved after each epoch, and training progress was logged every 100 iterations. A fixed random seed was set to ensure reproducibility of results. To accelerate computation, CUDA and cuDNN were enabled. Furthermore, model parameters were initialized by loading weights from a pre-trained model, thereby improving convergence speed and providing a more favorable starting point for fine-tuning.

### Methods

#### Methodological framework

**Fig. 3:**
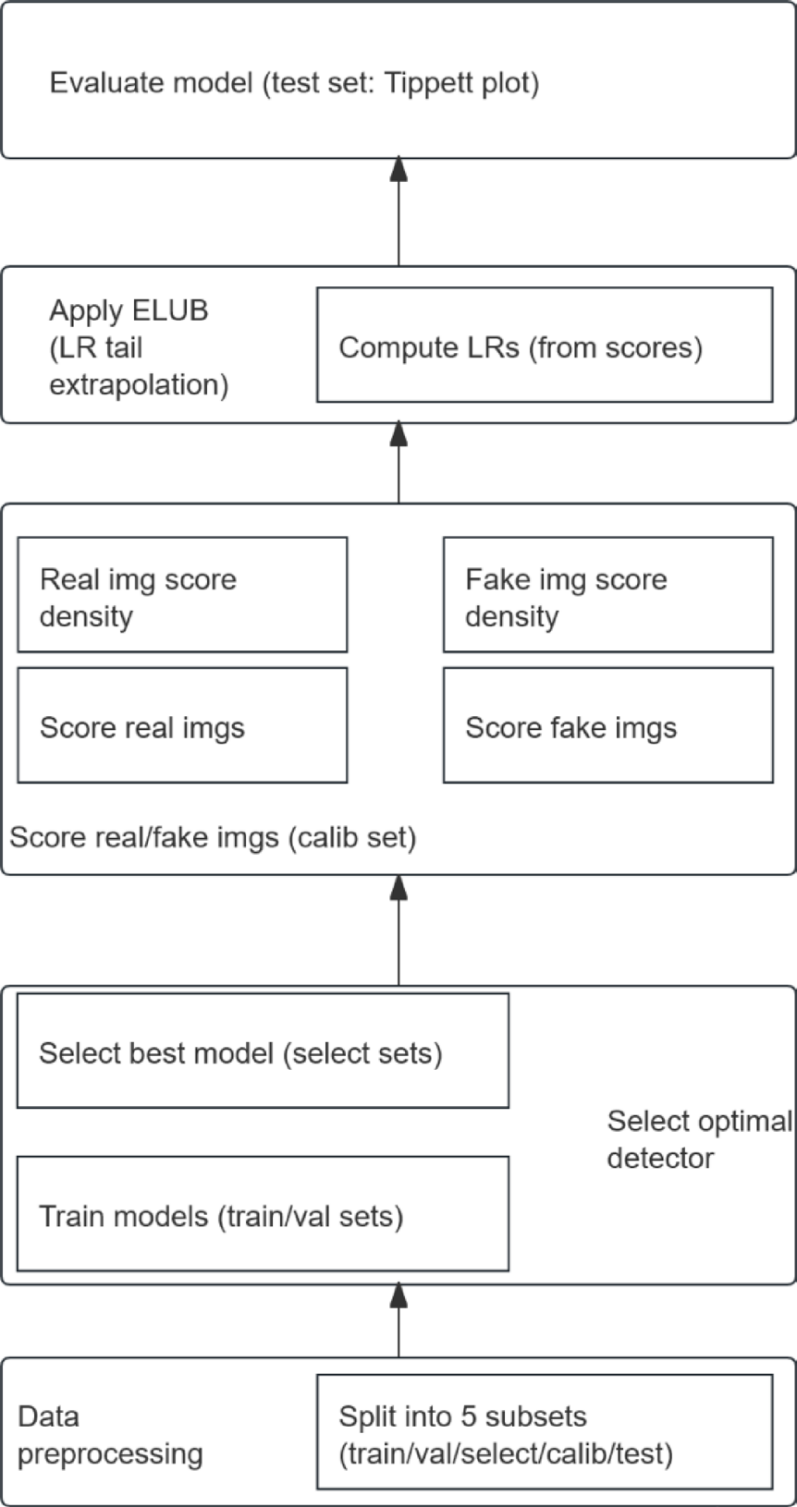
Methodological flowchart.

In the field of deepfake image detection research, the detection model can be interpreted as a mechanism for assigning a credibility score to the reality of images. The “confidence score indicating that an image is a deepfake,” produced by the model, corresponds directly to the output of this scoring mechanism. To develop a scientifically grounded identification model specifically tailored to the FF + + dataset, the research methodology is structured as Fig. 3.

The framework begins with data preprocessing, followed by the partitioning of the FF + + dataset into five non-overlapping subsets: training, validation, selection, calibration, and test sets. Subsequently, multiple models are trained on the training and validation sets, and the optimal deepfake detector is selected based on its performance on the selection set. Next, all real and fake images in the calibration set are scored, and the score distributions (i.e., score densities) for both classes (real and fake) are estimated. Following this, ELUB is applied to address tail extrapolation in LR estimation, and LR are computed from the resulting scores. Finally, the model is evaluated on the test set.

#### Scoring methodology

The majority of deepfake image detection models typically perform facial image reality assessment through a five-stage pipeline: input processing, backbone feature extraction, neck-level feature aggregation, head-level decision making, and confidence score generation^50^.

**Fig. 4:**
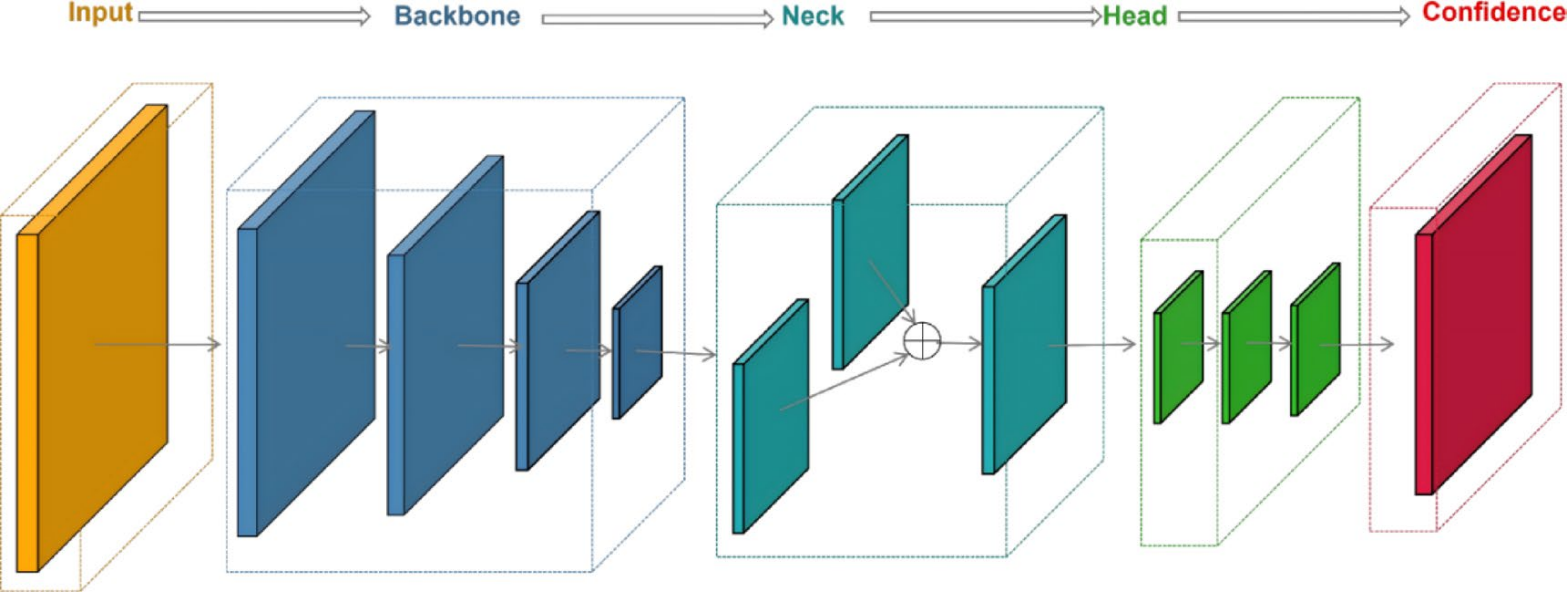
General architecture of deepfake detection models.

As illustrated in Fig. 4, the input layer crops facial regions to focus on salient features and removes irrelevant background. The backbone network extracts discriminative features using architectures like MobileNet, ResNet, or ViT, capturing differences between real and fake textures. The neck applies attention mechanisms and transforms feature maps into 1D vectors via pooling and flattening. The Head module refines features through fully connected layers, normalization, dropout, and Sigmoid activation. The output is a confidence score in[0,1], where higher values indicate deepfake likelihood and lower values indicate reality.

#### Model selection

In forensic-oriented scenarios, deepfake detection models are selected based on high overall accuracy and balanced performance across sub-categories.Overall accuracy is the primary metric, used to filter out poorly performing models early.Special attention is given to detection rates across different deepfake types to avoid blind spots.Balanced performance ensures reliability in identifying diverse deepfake techniques.When selection is inconclusive, a systematic elimination process removes models with low accuracy or weak performance in specific categories.

**Fig. 5:**
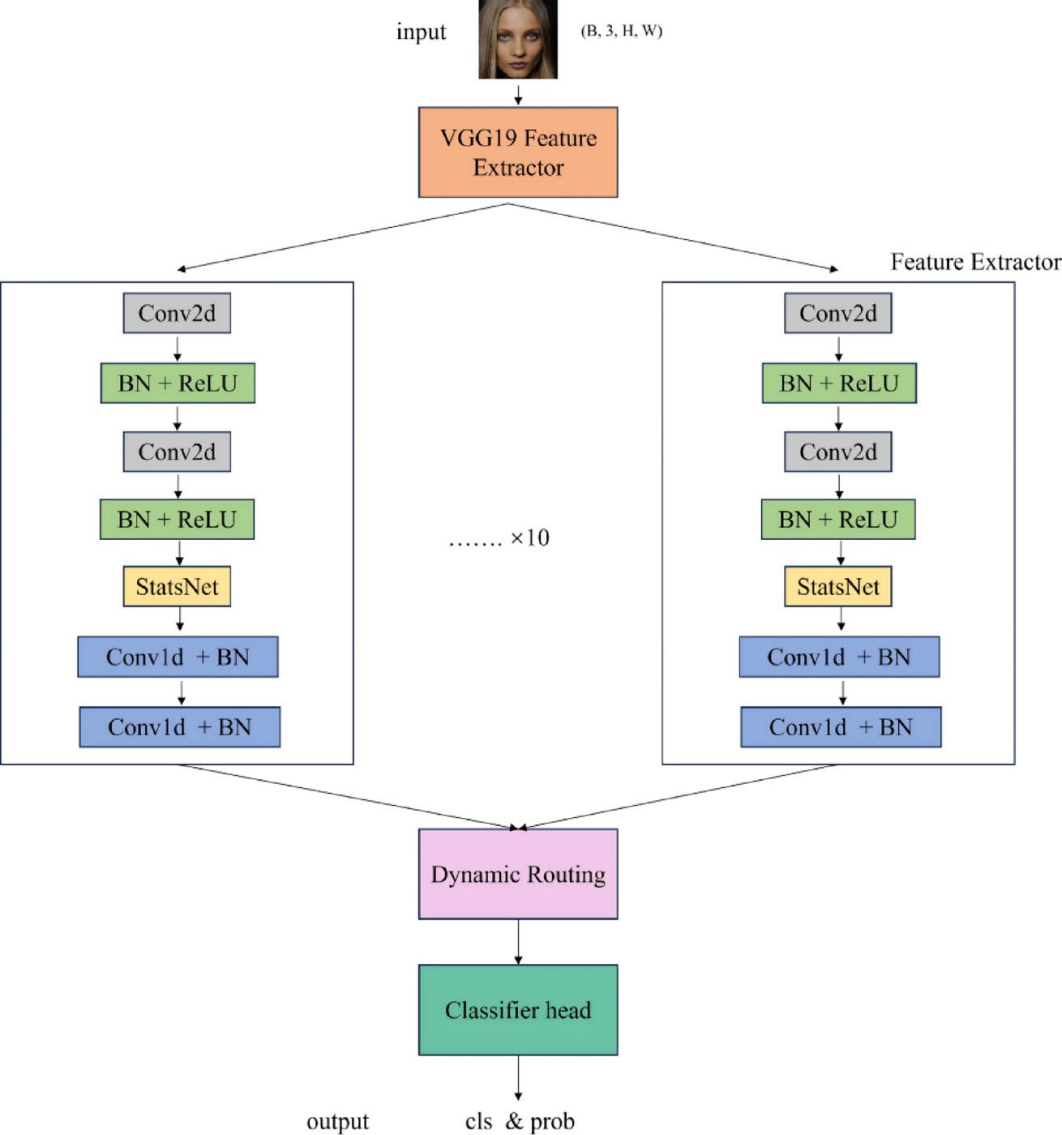
Network structure diagram of Capsule detector.

As shown in Fig. 5,the Capsule network deepfake face detector mentioned in 4.1 takes VGG19 as the basic feature extraction network and freezes the first 8 convolutional layers to retain general image features. Firstly, the input face image is transformed into high-level semantic features, and then the features are refined through 10 parallel Capsule branches. Each branch successively performs convolution operations, batch normalization, and StatsNet statistical feature extraction to capture local and global discriminative patterns. Subsequently, the dynamic routing mechanism (with 2 iterations) is utilized to achieve information aggregation among Capsules, effectively fusing the high-dimensional representations output by multiple branches. Finally, a classifier with Sigmoid activation outputs the probability distribution of “real” or “fake”, completing the task of discriminating deepfake faces.

1 $$Los{s_{capsule}} = \sum\nolimits_{k = 0}^{N - 1} {CrossEntropy({y_k},t)}$$

Formula (1) defines the loss function of the Capsule network, which is fundamentally a summation of cross-entropy losses across multiple parallel branches. Specifically, Loss_Capsule_ represents the overall loss, N denotes the total number of Capsule branches, y_k_ signifies the predicted output from the k-th Capsule branch, t stands for the ground-truth label, and CrossEntropy(·) refers to the standard cross-entropy loss function. During training, this loss is backpropagated through the network to update the weights of all trainable layers, enabling effective model optimization.

#### LR model based on scores

The probability density curves of two data types effectively capture the distribution characteristics of deepfake scores associated with real and deepfake images. The deepfake scores generated by a detection model are continuous values ranging from 0 to 1. For real images, most scores tend to cluster toward the lower end of the spectrum, although a small proportion may exhibit higher scores due to artifacts or model uncertainty. Similarly, for deepfake images, while scores generally concentrate near the upper end, some instances may yield lower scores, particularly in cases of high-quality deepfake or partial manipulation. Consequently, it is not feasible to rely solely on a fixed threshold to definitively classify an image as “real” or “fake.” Instead, the continuous nature of score distributions is best represented and analyzed through probability density curves.

The score based LR is computed as the ratio of these two density distributions and is a widely accepted measure in forensic science for quantifying the strength of evidential support. Specifically, it is defined as the ratio of the probability density of observing a given score under the prosecution’s hypothesis to the probability density of observing the same score under the defense’s hypothesis:

- Prosecution Hypothesis (Hp): The image is a deepfake.
- Defense Hypothesis (Hd): The image is real.

The LR thus provides a principled, probabilistic assessment of how much more likely the observed evidence is under one hypothesis compared to the other, forming a cornerstone of evidential evaluation in forensic decision-making.

2 $$SLR = \frac{{P(score|{H_p},I)}}{{P(score|{H_d},I)}}$$

Among formula(2), I denotes the background information, which encompasses the contextual knowledge or prior assumptions relevant to the analysis. Hp represents the prosecution’s hypothesis (the image is deepfake), and Hd denotes the defense’s hypothesis (the image is real). The “score” refers to the confidence score output by the deepfake detection model, reflecting the degree of suspicion associated with the input image. P(score|Hp, I) and P(score|Hd, I) denote the probability density functions of the observed score under the respective hypotheses. The LR has a clear probabilistic interpretation: for a given score, it quantifies the relative support for Hp compared to Hd. Specifically, if the score based LR (SLR) is significantly greater than 1, the evidence strongly favors Hp over Hd. Conversely, if the SLR is significantly less than 1, the evidence favors Hd over Hp.

In the evaluation of LR models within forensic science, core performance metrics and associated graphical tools recommended in forensic LR validation frameworks are utilized—including the log-likelihood ratio cost (C_llr_) as a key metric, along with DET plots, Tippett plots, and ECE plots as supporting graphical tools. These collectively reflect the model’ s primary performance characteristics (accuracy, discrimination, and calibration) to assess its reliability in forensic scenarios.

DET plots (Detection Error Tradeoff plots) visualize the trade-off between false positive and false negative rates, with curves closer to the origin indicating stronger discriminative power^51^. Tippett plots present the cumulative distribution functions of log-likelihood ratios (log-LRs) for real and deepfake samples, where greater separation between the two distributions reflects better ability to distinguish between hypotheses^52^. The Empirical Cross-Entropy (ECE) is a prior-dependent performance metric that quantifies calibration by comparing predicted probabilities with actual outcomes across bins, with lower values indicating better-calibrated models^53^. The log-likelihood ratio cost (C_llr_) serves as a summary metric of ECE (at equal prior probabilities of 0.5), quantifying the average information loss due to deviation from ideal log-LRs^54^. Together, these performance metrics and corresponding graphical representations assess the core primary performance characteristics (discrimination and calibration) of LR models, supporting the evaluation of their reliability in forensic analysis.

3 $$Cllr = \frac{1}{2}(\frac{1}{{{N_p}}}\sum\limits_{i \in true - {H_p}}^{{N_p}} {{{\log }_2}} (1 + \frac{1}{{L{R_i}}}) + \frac{1}{{{N_d}}}\sum\limits_{j \in true - {H_d}}^{{N_d}} {{{\log }_2}} (1 + L{R_j}))$$

Among them, the formula for calculating the C_llr_ is shown in Eq. (3), where Np represents the total number of deepfake class samples, Nd represents the total number of real class samples, and LRi​and LRj​represent the LR values converted from the original scores of the deepfake class and real class obtained from the same LR system model, respectively.

ELUB addresses unreliable extreme LR values caused by tail extrapolation in forensic LR systems. It combines NBE evaluation with CMLR augmentation—extending tails using synthetic misleading LRs (lower for Hp, higher for Hd). Using a non-parametric approach, ELUB accounts for set size, discrimination, and performance to establish empirical LR bounds that limit extrapolation to statistically justifiable levels. This ensures that even with rare or misleading tail evidence, the LR system performs at least as well as the neutral system. By preserving this minimum performance, ELUB enhances the robustness and reliability of LR outputs in judicial decision-making^55^.A core metric underpinning ELUB is the Normalized Bayes Error-rate (NBE), defined in Eq. (4). This metric validates the reliability of LR systems when extrapolating to extreme evidence strengths, as proposed in standard forensic LR validation frameworks.

4 $$NBE = \frac{{EU(neutral)}}{{EU(LRsystem)}} = \frac{{{P_{Hp1 \le L{R_{th}}}} + L{R_{th}} \times {P_{Hd1L{R_{th}}}}}}{{\frac{{{N_{CMLR}} + {n_{Hp,LR \le L{R_{th}}}}}}{{{N_{CMLR}} + {n_{Hp}}}} + L{R_{th}} \times \frac{{{N_{CMLR}} + {n_{Hd,LRL{R_{th}}}}}}{{{N_{CMLR}} + {n_{Hd}}}}}}$$

Equation (4) defines the NBE, a key metric for validating the reliability of LR systems when extrapolating to extreme evidence strengths (e.g., very high/low LR values), as proposed in forensic LR validation frameworks (e.g., the ELUB method). The numerator quantifies the expected utility of a “neutral system” (i.e., a system that provides no evidence beyond prior odds): P_Hp,1_≤LR_th_ represents the proportion of Fake (Hp) samples with LR ≥ 1 (supporting the Hp proposition), while P_Hd,1_> LR_th_ is the proportion of Real (Hd) samples with LR<1 (supporting the Hd proposition); LR_th_ denotes the decision threshold for LR. Together, these terms capture the baseline evidence strength expected without an LR system. The denominator calculates the expected utility of the calibrated LR system after incorporating Critical Misleading LRs (CMLRs)—synthetic extreme LR values added to simulate tail-end extrapolation: N_CMLR_ is the number of CMLRs, n_Hp, LR_≤LR_th_ (or n_Hd, LR> LRth_) is the count of original Hp (or Hd) samples with LR≤LR_th_ (or LR> LRth), and n_Hp_(or n_Hd_)is the total number of original Hp (or Hd) samples. The fractional terms adjust the probability of extreme LR values to account for CMLR-induced tail distribution changes.

The NBE ratio compares these two utilities: an NBE ≤ 1.1 (a commonly accepted threshold in forensic science) indicates that the LR system remains more reliable than a neutral system even when extrapolating to extreme evidence strengths, justifying the use of an ELUB to constrain LR values within interpretable, robust bounds.

## Experiments

### Model Selection

**Fig. 6:**
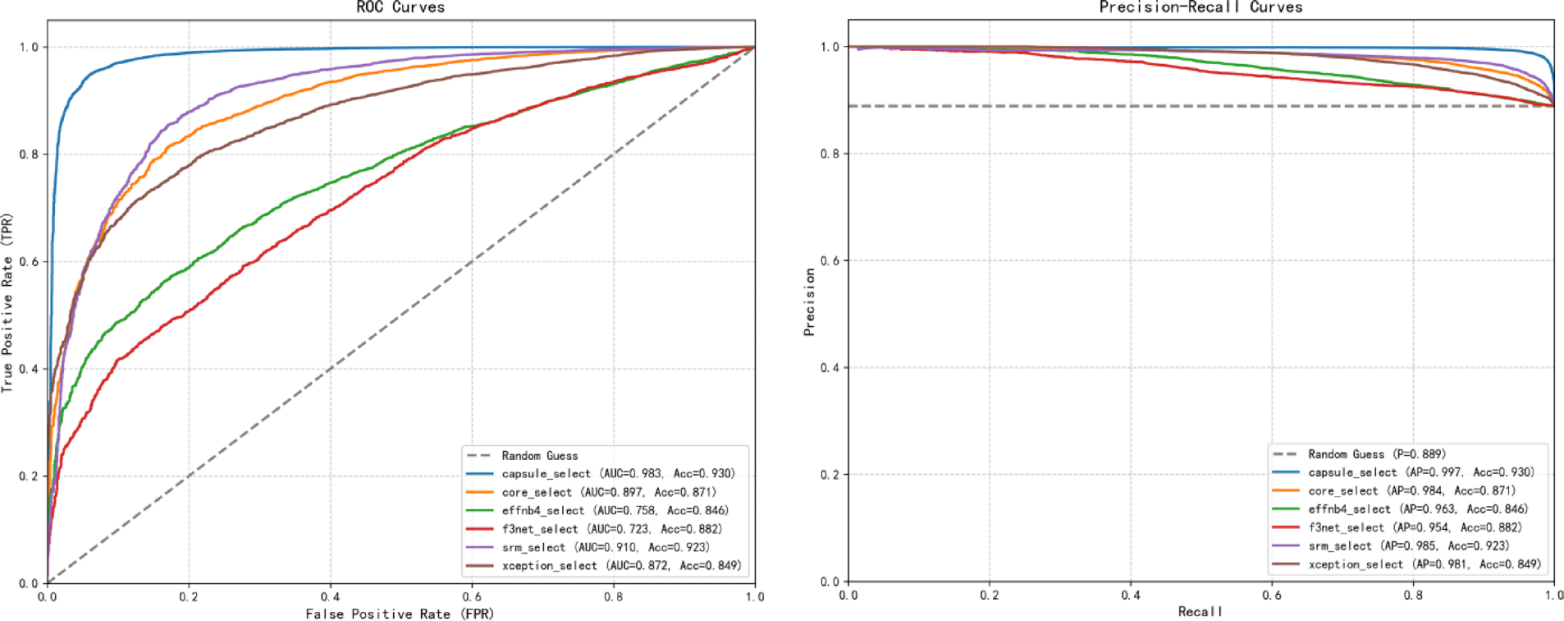
ROC curves (left) and AP curves (right) of each detection model on the FF + + selectiondataset.

Six deepfake image detection models—Capsule [6–8], Core [9], EfficientNetB4 (hereinafter referred to as EffNB4) [10], F3Net [11], SRM [12], and Xception [13]—were selected for initial training. During the training process, each model was trained on the same training set and validated using the same validation set, with iterative training cycles and hyperparameter optimization conducted to ensure consistent evaluation conditions. The performance results of the individual models are summarized in Fig. 6.

Based on Fig. 6, the Capsule detector is selected based on its balanced performance across overall accuracy and sub-category detection rates on the select subset. On the left ROC curve, its curve is closest to the top-left corner and achieves the highest AUC (0.983), indicating a good balance between true positive and false positive rates, and strong discrimination between real and deepfake samples. On the right Precision-Recall curve, it also performs best with an Average Precision (AP) of 0.997, maintaining high precision as recall increases—critical for minimizing false alarms in deepfake detection. It also achieves the highest accuracy (0.930) on this subset, as shown in the legend. Other models show lower AUC, AP, or accuracy. Overall, the Capsule detector exhibits more consistent and reliable performance across the evaluated metrics.

**Fig. 7:**
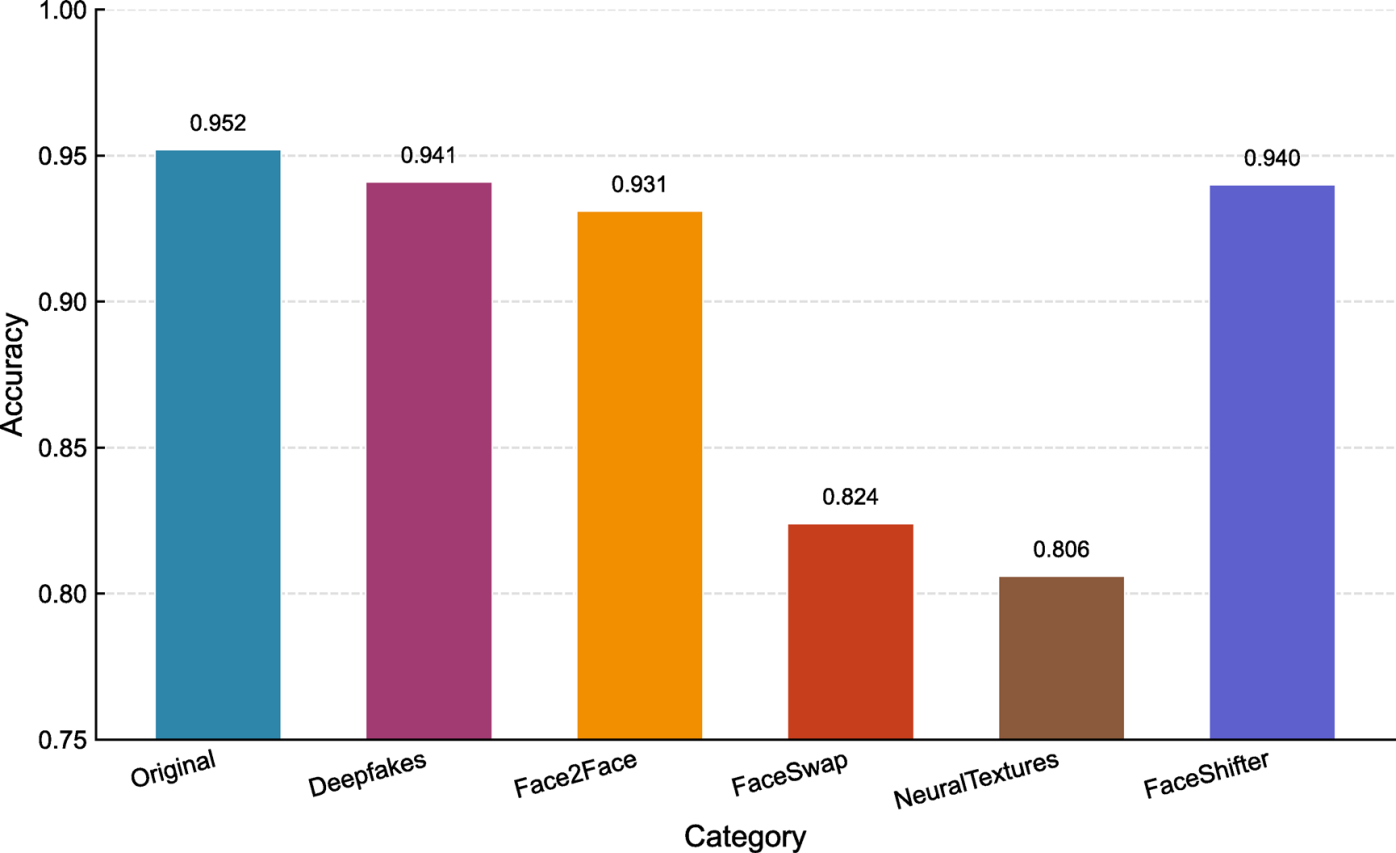
Accuracy of Capsule detector on FF + + dataset subcategories.

Figure 7 shows the accuracy of the Capsule Detector in various categories, further demonstrating its effectiveness. For real videos, its accuracy rate is as high as 0.952, confirming its robustness in recognizing real facial content. For the false subcategories, its results remain strong: the accuracy rates for Deepfakes (0.941), Face2Face (0.931), and FaceShifter (0.940) are all close to 0.95. Even in more challenging cases - FaceSwap (0.824) and NeuralTextures (0.806), where the manipulations are visually subtle and more difficult to detect, the model still achieves an accuracy rate above 0.80, which is considered feasible in practical applications.

In conclusion, the Capsule Detector not only exhibits competitive performance in key metrics among the evaluated models (as evidenced by the highest ROC-AUC and PR-AP scores on the FF++ “selected” subset), but also demonstrates consistently reliable accuracy across all data subcategories. These findings collectively establish the Capsule Detector as the most appropriate choice for DeepFake detection on the FF + + dataset.

### Probability Density Estimation

**Fig. 8:**
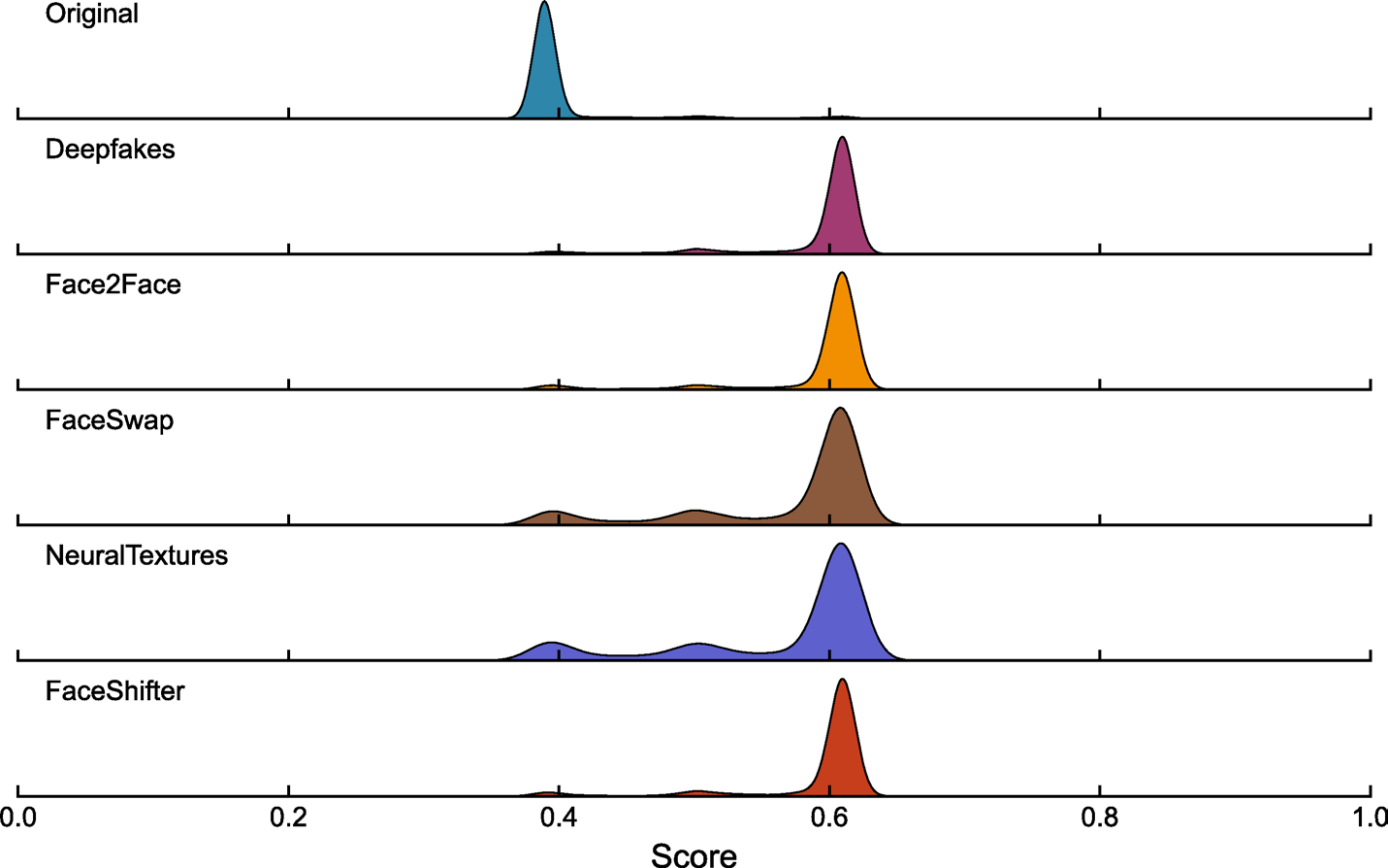
Score distributions of real images and subset images from the FF + + dataset.

Prior to performing probability density estimation, it is essential to evaluate whether the score distributions across sub-classes are homogeneous and whether the peak-valley effect may interfere with the visualization of individual sub-class distributions. Therefore, before estimating the score probability density for the FF + + dataset, a ridgeline plot is utilized to examine the histogram distribution of each sub-class. This method effectively illustrates the central tendency, dispersion, and shape of the data distributions, as shown in Fig. 8.

Figure 8 reveals that the score distribution of the Original (real class) is tightly concentrated around 0.4, exhibiting a homogeneous and well-defined pattern. In contrast, all fake sub-classes—Deepfakes, Face2Face, FaceSwap, NeuralTextures, and FaceShifter—consistently peak near 0.6, with only minor differences in dispersion and shape across sub-classes.

The consistent central tendency of the fake sub-classes in the high-score region (~ 0.6), together with the distinct clustering of the real class in the low-score region (~ 0.4), demonstrates that the distributions within each main category (real or fake) are highly homogeneous. The subtle variations among individual fake sub-classes do not undermine the overarching bimodal structure of the overall score distribution.

Accordingly, it is justified to aggregate the sub-classes into two unified groups—real and fake—for probability density estimation. A single density function can effectively model the real class, while another captures the collective distribution of all fake sub-classes. This approach streamlines the modeling process by abstracting away negligible sub-class-level discrepancies, while faithfully retaining the essential distributional features of the two primary categories.

The optimal bandwidths were determined through a two-stage strategy—coarse search followed by fine search—combined with 3-fold cross-validation. To balance model fit and distribution alignment, a joint evaluation metric was employed, integrating the cross-validated log-likelihood.

5 $$L\left(\lambda\right)={\sum}_{i=1}^{n}{log}(\frac{1}{n\lambda}{\sum}_{j=1}^{n}K(\frac{{x}_{i}-{x}_{j}}{\lambda}+\epsilon))$$

In light of the verification findings,

6 $$bandwidt{h}_{real}=0.004$$

7 $$bandwidt{h}_{fake}=0.003$$

**Fig. 9:**
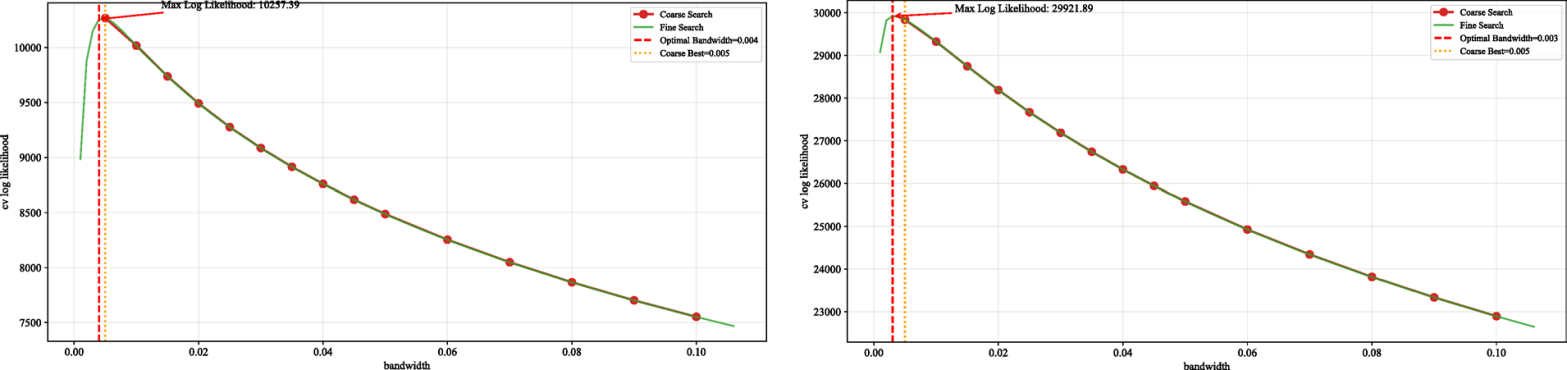
Bandwidth Search vs. Cross-Validated Log Likelihood.

In Formula 5, n is the sample size, λ the bandwidth parameter to be optimized, K(·) the Gaussian kernel function, and ε = 10e-10 a small constant ensuring numerical stability in logarithmic operations. Bandwidth optimization is illustrated in Fig. 9, which contains two subplots: the left for the real category and the right for the deepfake category. Red dots represent cross-validation log-likelihood values from the coarse search across different bandwidths, while the green curve shows the fine search trajectory near the coarse-stage optimum. The red dashed line indicates the final optimal bandwidth (0.004 for real, 0.003 for deepfake), and the yellow dashed line marks the coarse-stage optimal value. Red text displays the maximum cross-validation log-likelihood at the optimal bandwidth—10237.39 for the real class (at 0.004) and 29921.89 for the deepfake class (at 0.003), with higher values indicating better fit to the sample distribution. Using a two-stage strategy—coarse search to identify an approximate range and fine search to refine precision—the optimal bandwidth is efficiently determined without excessive computation. Final results are given in Formulas 6 and 7, showing optimal bandwidths of 0.004 for the real category and 0.003 for the deepfake category.

**Fig. 10:**
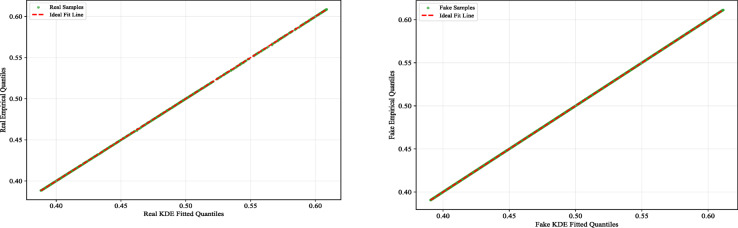
QQ Plots of KDE fit for real/fake samples.

Figure 10 shows the QQ plots of kernel density estimation (KDE) fits for real and fake samples. In both groups, the empirical quantiles (green points) closely follow the red dashed “Ideal Fit Line,” indicating strong agreement between the sample score distributions and the KDE-derived quantiles using the optimized bandwidths (0.004 for real, 0.003 for deepfake). This close alignment confirms that the selected bandwidths produce a well-fitted KDE model.

We obtained the distribution of scores for the two types of images based on bandwidth and Gaussian kernel function. For real images, the scores are mostly concentrated in a narrow range between 0.4 and 0.45. For deepfake images, the scores sharply increase around 0.6, also showing a narrow distribution. The clear separation between the two distributions provides evidential support for the model’s discriminative ability. This distinct bimodal pattern indicates that the model has effectively captured discriminative features between real and deepfake images, enabling relatively consistent score assignment for the two categories. The density distribution model derived from the calibration set is saved in PKL format for subsequent ELUB optimization.

### Optimization of the LR Model

**Fig. 11:**
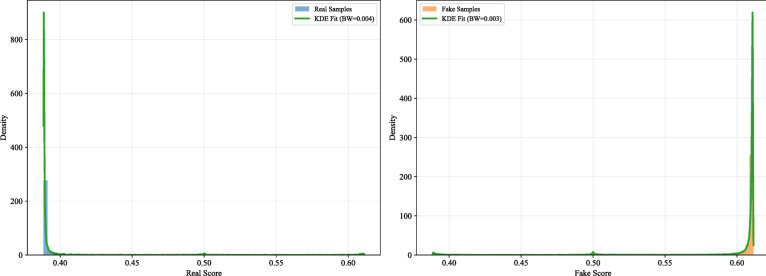
log₁₀(LR) distribution of the calibration set.

We saved the density model and applied it to the calibration set to compute the log-likelihood ratio (log₁₀(LR)) for each score. As shown in Fig. 11, the majority of LR values fall within the − 3 to + 3 range. Nevertheless, a small number of data points extend into the 3–10 range: although sparse, these values are non-negligible and exhibit a “weak but abnormal” pattern that raises concerns about their origin and reliability.

**Fig. 12:**
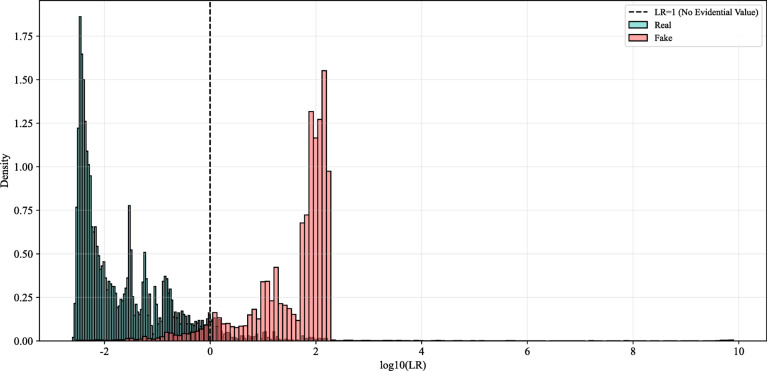
Results of probability density fitting.

Figure 12 reveals that real and faked samples exhibit distinct score peaks near 0.4 and 0.6, respectively. In the intervening interval (0.4, 0.6), a low-density “long tail” emerges despite minimal empirical support. Due to the smoothing mechanism inherent in KDE, this data-sparse region is assigned non-zero probability density, which directly contributes to the inflated LR values observed in Fig. 12.These extreme LR values are not indicative of actual sample prevalence but rather reflect a known limitation of KDE—its tendency to perform smooth extrapolation beyond the support of observed data. This behavior generates spurious density estimates in regions with little or no empirical basis, leading to unreliable high-magnitude likelihood ratios.

Given this risk of overestimation in the distribution tails due to insufficient data coverage, the ELUB method becomes critically important. By imposing empirically derived upper and lower bounds on LR values, ELUB effectively truncates extrapolated extremes lacking sufficient sample support, thereby enhancing the robustness and forensic validity of downstream decision-making.

**Fig. 13:**
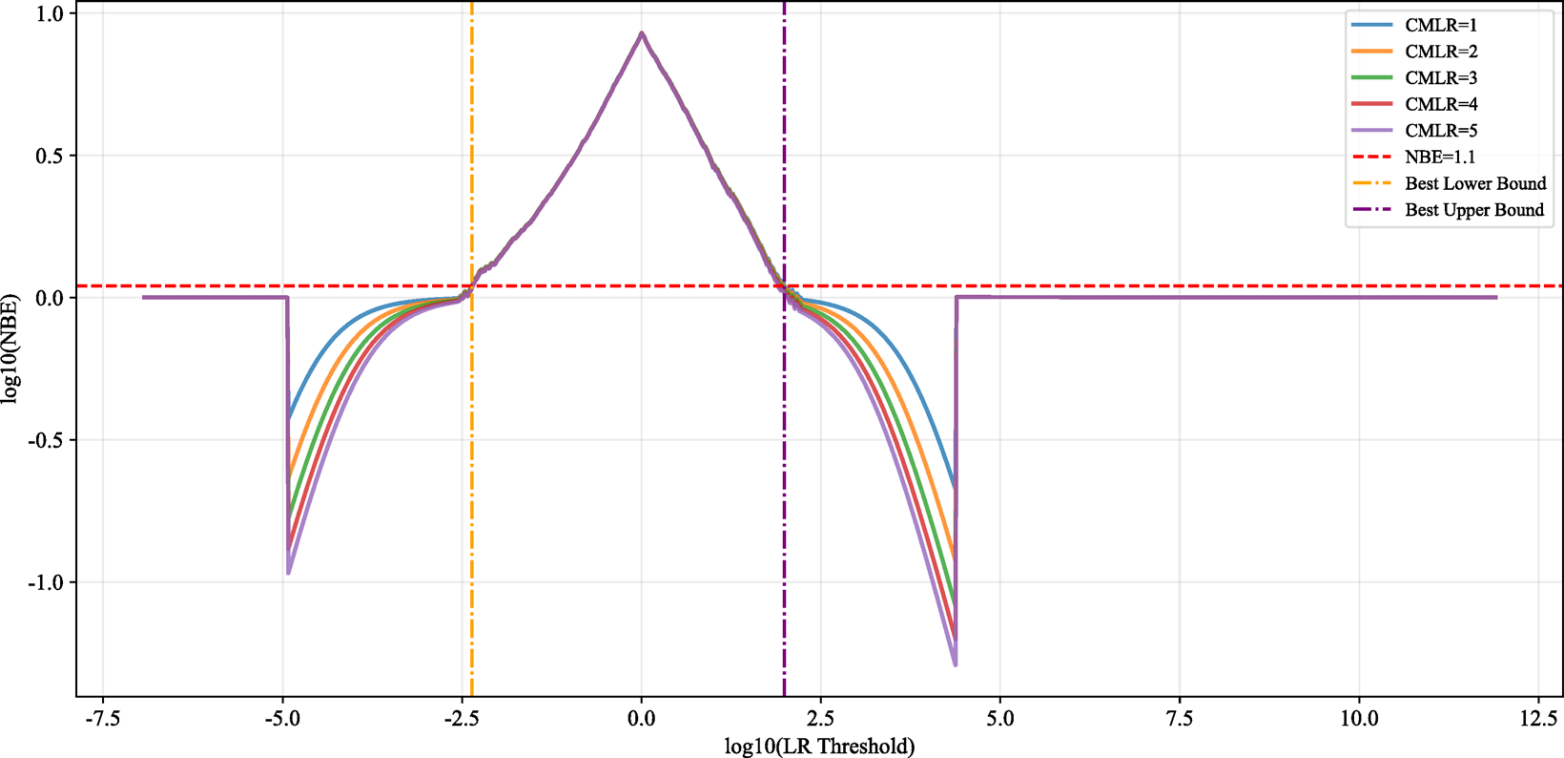
NBE Curves by CMLR Count.

Figure 13 visualizes the ELUB method by comparing NBE curves for different counts of Consequential Misleading LRs (CMLR, 1 to 5). It identifies reliable LR system bounds and addresses unreliable extreme LRs caused by tail extrapolation due to limited data. The x-axis shows log₁₀(LR Threshold), the logarithmic LR decision threshold—essential for handling the wide LR range (e.g., 10⁻⁷.⁵ to 10¹².⁵)—enabling clear visualization of high-risk tail regions. LRs above the threshold support the prosecution hypothesis (fake samples); those below support the defense hypothesis (real samples). The y-axis shows log₁₀(NBE), with a red dashed line at log₁₀(1.1) indicating the performance threshold. When NBE curves (colored solid lines) rise above this line, the LR system performs worse than a neutral system, marking unreliable LR ranges. The optimal ELUB bounds (yellow and purple dashed lines) define the transition between reliable and unreliable regions.

**Table 2:** CMLR optimization summary.

CMLR no.	ELUB Lower	ELUB Upper	Extreme Ratio	C_llr_
1	−2.3634	1.9933	0.3570	0.0358
2	−2.3606	1.9791	0.3726	0.0358
3	−2.3577	1.9344	0.4220	0.0360
4	−2.3539	1.9117	0.4525	0.0361
5	−2.3502	1.9064	0.4599	0.0361

Table 2 shows CMLR = 1 yields the lowest C_llr_. For CMLR ≥ 2, C_llr_ shows no meaningful improvement. As CMLR increases from 1 to 5, the lower bound rises slightly from − 2.3634 to −2.3502, and the upper bound decreases from 1.9933 to 1.9064, making the bounds more conservative. However, the proportion of extreme samples outside the bounds increases from 0.3570 (CMLR = 1) to 0.4599 (CMLR = 5), showing that higher CMLR counts unnecessarily exclude valid data, reducing utilization efficiency.

The ELUB bounds for CMLR = 1 (lower: −2.3634; upper: 1.9933) were selected as they effectively truncate unreliable tail LRs while preserving the most valid samples, balancing robustness and data efficiency. This optimized rule was then applied to the test sample LRs.

### Validation of the LR model

#### Primary performance characteristics

The LR system is developed using training, validation, selection, and calibration datasets, and its performance is evaluated on an independent test set through the Tippett plot, DET plot, and ECE curve [56].

**Fig. 14:**
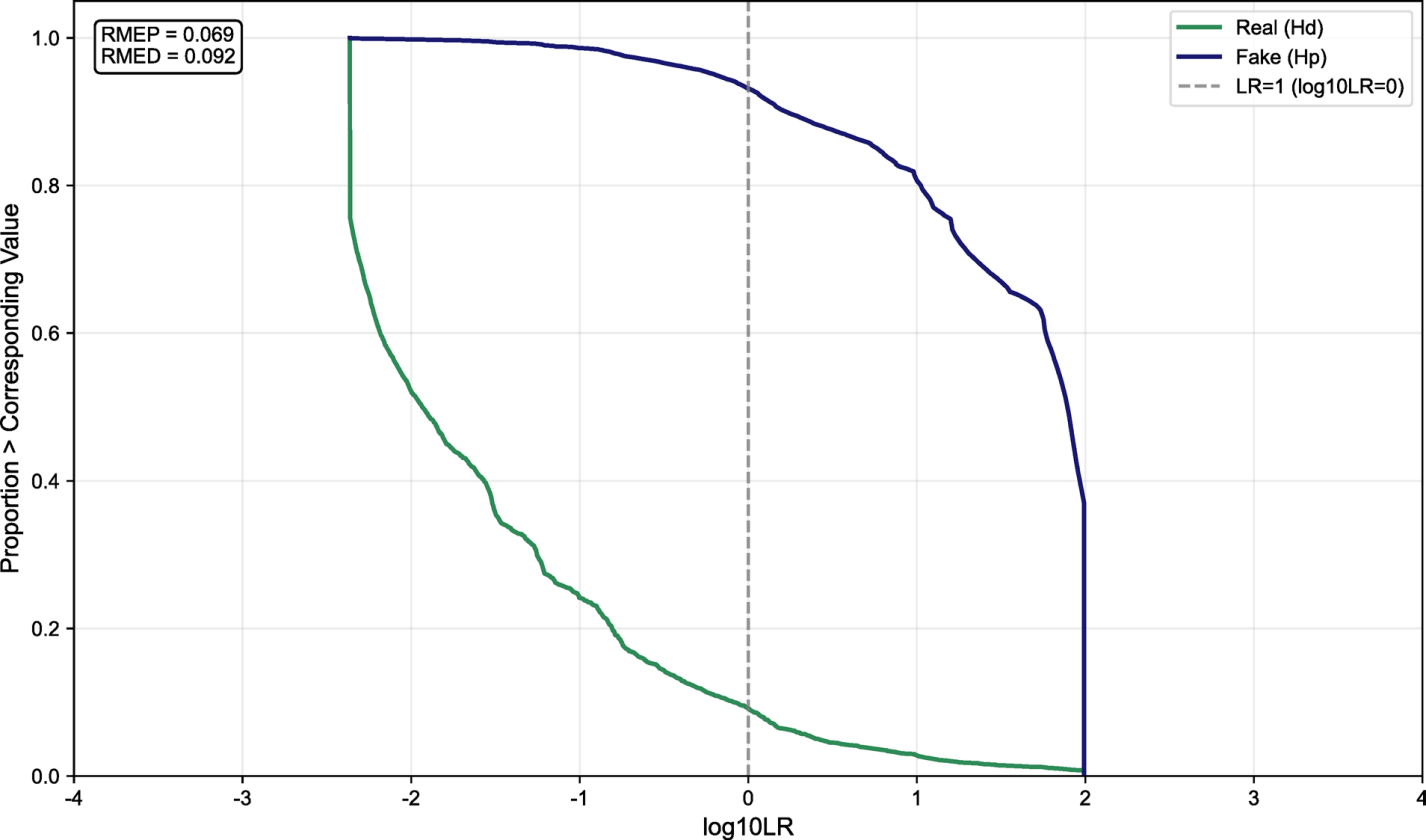
Tippett plot.

The Tippett plot serves as a fundamental tool in forensic science for quantifying the discriminative power of LR systems. It displays the cumulative distribution of log_10_(LR) values under Hp (the proposition that a sample is fake) and Hd (the proposition that a sample is real). As shown in Fig. 14, the decreasing trend of both real and fake curves indicates that higher log_10_(LR) values are increasingly rare across both classes. ELUB processing constrains log_10_(LR) within (–3, 3), limiting LR values to the range 10^− 3^–10³. This constraint mitigates the impact of extreme values while aligning with common practices for interpretable and stable evidence strength reporting in forensic-related research. Critically, the results show RMEP = 0.069 and RMED = 0.092—indicating that only 6.9% of fake samples yield LR < 1 under Hp, and only 9.2% of real samples yield LR > 1 under Hd. Such strong separation between hypotheses demonstrates high discriminative accuracy, with minimal misalignment in evidential direction. These findings confirm that the LR system achieves good distinction between real and fake samples on the test set.

**Fig. 15:**
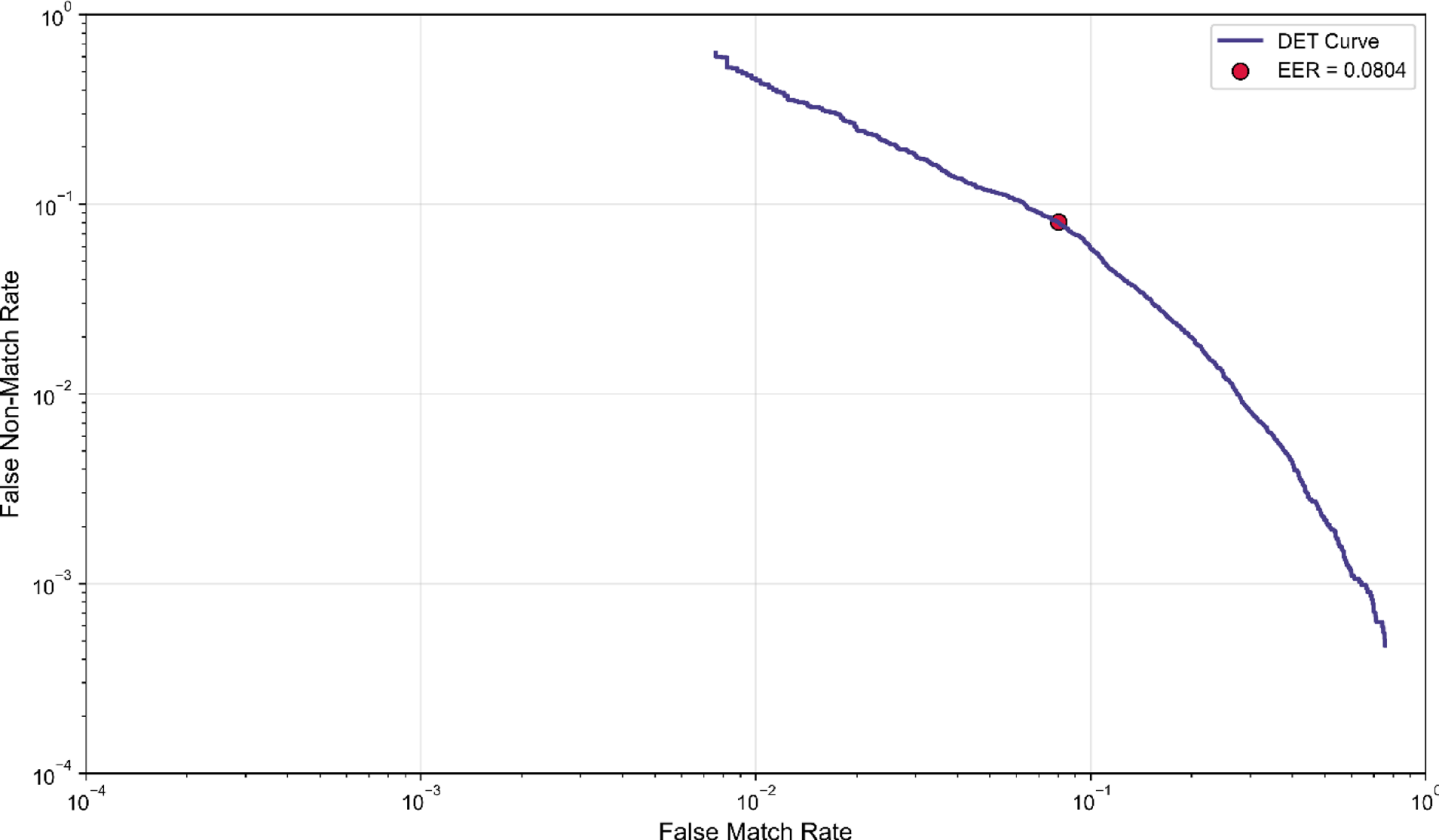
DET plot.

The DET plot evaluates the system’s error control by illustrating the trade-off between false match rate (FMR) and false non-match rate (FNMR) across decision thresholds. In Fig. 15, logarithmic scaling enables precise visualization of performance in low-error regimes, which is essential for forensic applications. The equal error rate (EER = 0.0804) represents the point where FMR and FNMR intersect, reflecting balanced bidirectional error performance. A low EER value, combined with the curve’s proximity to the origin, indicates consistently low error rates across thresholds. This demonstrates robust error management and supports the system’s reliability in practical forensic contexts.

**Fig. 16:**
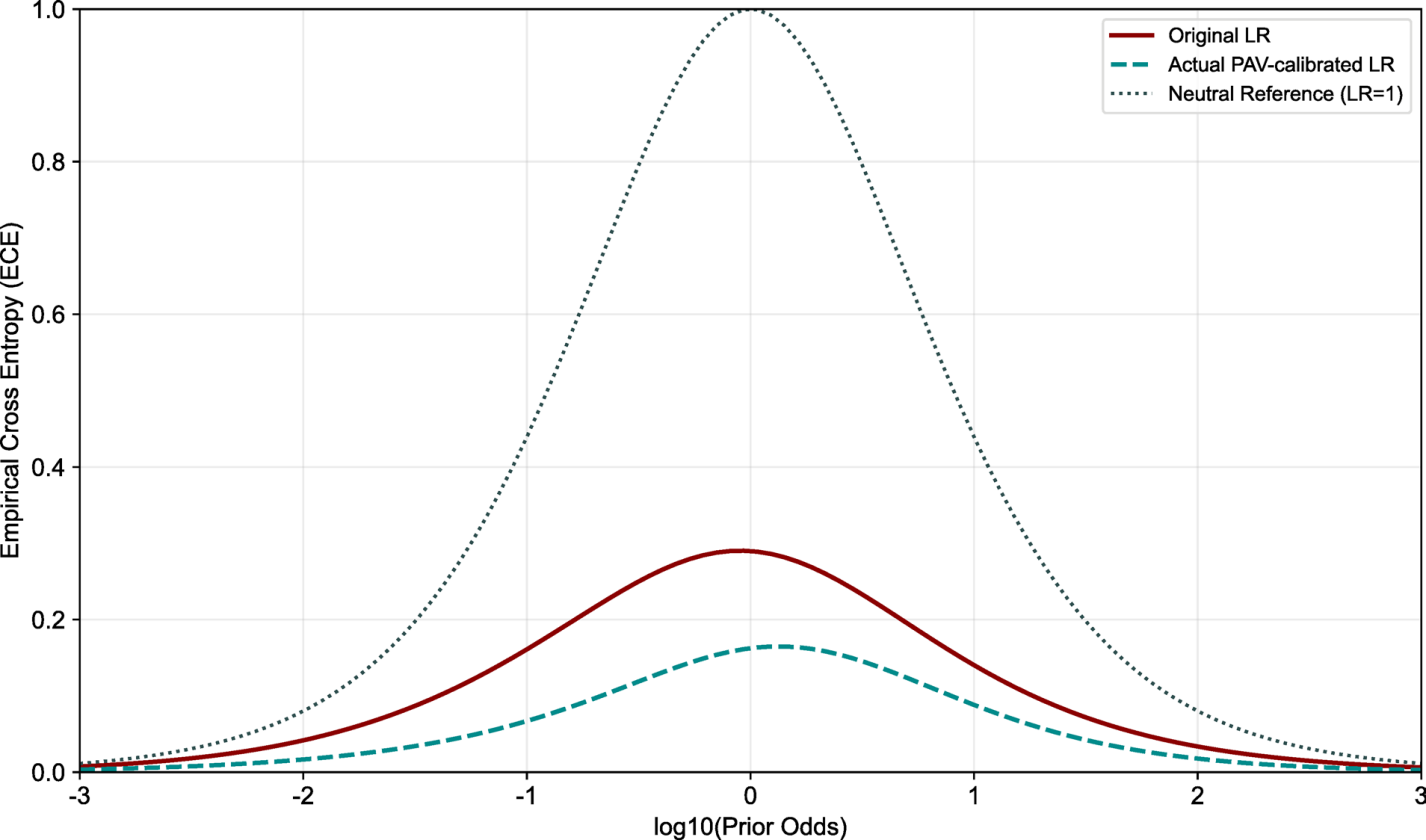
ECE plot.

Calibration reliability is assessed using the ECE curve, which measures the mismatch between predicted LR-based evidential strength and empirical evidential strength across prior odds (P(Hp)/P(Hd)). As shown in Fig. 16, the Pool Adjacent Violators (PAV) algorithm—by design to separate the calibration performance from the discriminative power—effectively reduces calibration loss The uncalibrated system yields a C_llr_ of 0.2899, while the theoretical minimum ($C_{llr}^{\min }$ = 0.1625)—a fixed value reflecting the data’s inherent discriminative power—remains unchanged.

In summary, the evaluation of primary performance characteristics reveals that the constructed LR system exhibits favorable discriminative accuracy, effective error control, and acceptable calibration performance on the independent test set.

#### Secondary performance characteristics

**Fig. 17:**
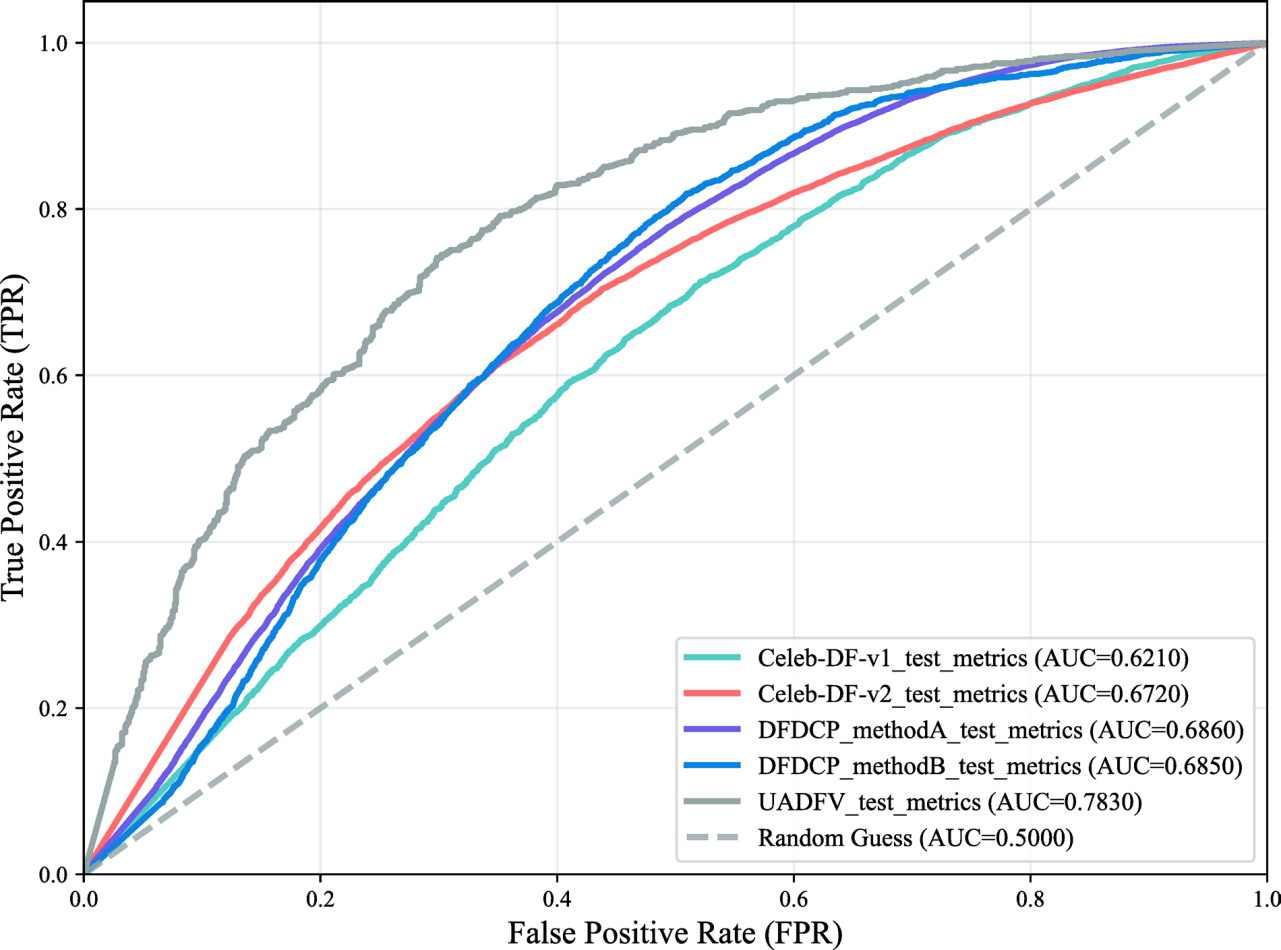
ROC curve of the LR system evaluated on unseen datasets.

The LR system developed in this study is evaluated on five unseen deepfake datasets: Celeb-DF-v1, Celeb-DF-v2, DFDC, DFDCP, and UADFV. All samples are passed through the detection model to generate scores, which are used in a KDE framework with bandwidths of 0.004 (real, Hd) and 0.003 (fake, Hp) to compute LRs. The LRs are optimized using ELUB, and performance is assessed via ROC curves (Fig. 17).

Results show the system achieves basic discrimination across all datasets, with AUCs ranging from 0.621 (Celeb-DF-v1) to 0.783 (UADFV), confirming its ability to distinguish real from fake. The high AUC on UADFV and its ROC curve near the top-left corner indicate strong separation and good generalization. Similar AUCs for DFDCP_methodA (0.686) and DFDCP_methodB (0.685) show stable performance across related variants, supporting moderate generalization within similar domains.

However, performance varies significantly. Celeb-DF datasets yield lower AUCs (v1: 0.621, v2: 0.672), with ROC curves far from ideal, indicating poor true-positive vs. false-positive trade-offs and higher decision risk. This is due to distributional mismatch: UADFV resembles the calibration set (FaceForensics++), while Celeb-DF has different generation patterns and feature distributions, weakening LR discriminability.

Operationally, the system demonstrates relatively reliable performance on UADFV and acceptable performance on DFDCP for low-stakes preliminary screening, but it does not perform well on Celeb-DF, where results are inconsistent and unsuitable for practical deployment. This underscores a fundamental issue in forensic AI: deepfake detectors have limited generalization capabilities. As evidenced by our reliance on FaceForensics++, transferring low-resource systems to dissimilar datasets without validation poses a risk of drawing incorrect conclusions. In sensitive application scenarios like forensic practice, low-resource-based evaluations should incorporate careful domain adaptation and clear performance boundary analysis.

## Conclusion

This paper presents a score-based LR system for forensic identification of deepfake images, built on the FaceForensics++ (FF++) dataset. Data are partitioned at the video level into training, validation, selection, calibration, and test sets to prevent data leakage and overfitting. From six candidate models—including Capsule, Core, and EfficientNetB4—the Capsule detector is selected as the core score generator due to its high AUC (0.993) and consistent per-class accuracy (≥ 0.80). Score distributions for real and fake samples in the calibration set are modeled using kernel density estimation (KDE), with optimal bandwidths determined via a two-stage search combined with 3-fold cross-validation (real: 0.004, fake: 0.003); QQ plots confirm good fit. To address unreliable extreme LR values from tail extrapolation, the ELUB method is applied, setting bounds at − 2.3634 to 1.9933 (CMLR = 1), effectively removing unsupported extremes.

Evaluation shows strong performance on the FF + + test set: low misleading evidence rates (RMEP = 0.069, RMED = 0.092), low equal error rate (EER = 0.0804), and well-calibrated decisions (C_llr_ reduced from 0.2899 to 0.1625). On five unseen datasets (Celeb-DF-v1/v2, DFDCP_methodA/B, UADFV), AUCs range from 0.621 to 0.783—above chance level—with best performance on UADFV (0.783), stable results on DFDCP (0.686, 0.685), and weaker generalization on Celeb-DF (0.621, 0.672), where ROC curves deviate from ideal shape.

Performance depends on the similarity of data distributions, tending to be strong when aligned with calibration data but degrading under domain shift. This finding suggests that cross-domain adaptation may be a key challenge for practical implementation. The framework may provide a potentially useful and interpretable framework for evidential assessment in scenarios with data distributions similar to FF++, while also providing insights for future improvements, including expanding calibration data coverage and integrating domain adaptation techniques to enhance generalization.

## Data Availability

The dataset supporting the findings of this study is publicly available at the GitHub repository of FaceForensics: https://github.com/ondyari/FaceForensics. The code implemented for this study can be accessed at: https://github.com/guotianli/Deepfakes-LR.
